# Ten tips for receiving feedback effectively in clinical practice

**DOI:** 10.3402/meo.v19.25141

**Published:** 2014-07-28

**Authors:** Ali H. Algiraigri

**Affiliations:** Community Health Sciences, University of Calgary, Calgary, AB, Canada

**Keywords:** feedback, self-assessment, self-awareness, career development, medical education

## Abstract

**Background:**

Despite being recognized as a fundamental part of the educational process and emphasized for several decades in medical education, the influence of the feedback process is still suboptimal. This may not be surprising, because the focus is primarily centered on only one half of the process – the teachers. The learners are the targets of the feedback process and improvement needs to be shifted. Learners need to be empowered with the skills needed to receive and utilize feedback and compensate for less than ideal feedback delivery due to the busy clinical environment.

**Methods:**

Based on the available feedback literature and clinical experience regarding feedback, the author developed 10 tips to empower learners with the necessary skills to seek, receive, and handle feedback effectively, regardless of how it is delivered. Although, most of the tips are directed at the individual clinical trainee, this model can be utilized by clinical educators involved in learner development and serve as a framework for educational workshops or curriculum.

**Results:**

Ten practical tips are identified that specifically address the learner's role in the feedback process. These tips not only help the learner to ask, receive, and handle the feedback, but will also ease the process for the teachers. Collectively, these tips help to overcome most, if not all, of the barriers to feedback and bridge the gaps in busy clinical practices.

**Conclusions:**

Feedback is a crucial element in the educational process and it is shown that we are still behind in the optimal use of it; thus, learners need to be taught how to better receive and utilize feedback. The focus in medical education needs to balance the two sides of the feedback process. It is time now to invest on the learner's development of skills that can be utilized in a busy day-to-day clinical practice.

Feedback is a dynamic process that involves the senders (typically the teachers) and the receivers (typically the students) to confirm positive behaviors (by encouraging repetition) and correct negative ones (by encouraging a change) (1). Despite been recognized as an integral part of the educational process (2) and emphasized in medical education for several decades (3–10), little has been done to empower the receivers (learners) with the skills to accept and use feedback effectively (5). This may be a reflection of the hierarchical medical environment that promotes a one-way flow of information from teacher to learner. As such, the literature is rich in describing the mechanism of feedback and how to deliver it (11). Furthermore, different models have been published and new ones continue to evolve that center on the teacher's role in providing effective feedback to enhance medical performance (12). In addition, faculty development courses and workshops have been implemented to teach how to deliver effective feedback (13). Despite all this, the results of feedback are still not optimal and sometimes even disappointing (3, 14–16).

Self-assessment is the first step in the feedback process and represents the person's ability to self-assess for a particular task. Although, it may sound easy to do, the literature tells us that we are poor at this skill (17). Indeed, it is recognized as one of the hardest skills, as described by Benjamin Franklin in 1750. A recent study explored a few reasons why we are not good self-assessors; these represent cognitive (information deficits and memory bias), sociobiological (unrealistic optimism), and social factors (inadequate feedback from others) (18). Improving self-assessment can identify weaknesses and improve acceptance of feedback, leading to positive changes in behavior and performance.

The importance of self-assessment to feedback is becoming more crucial with the current shift in the medical education paradigm toward competency-based curriculums, where learners are expected to achieve specific milestones enabling them to practice as competent physicians in their field (19). Effective feedback from both perspectives, delivering and receiving, helps learners to achieve those milestones. Simulation-based medical education (SBME) gained popularity and has been implemented in most medical schools and feedback is now considered to be a critical step in SBME (20).

Numerous barriers have been identified that prevent delivering effective feedback, including medical educators’ unfamiliarity with the feedback, timing and place constraints, and concerns with breaking the educational relationship between teacher and learner with negative feedback (21). In addition, the educational environment plays an important role in the process to enhance or limit the feedback process. On the other hand, there are several learners’ barriers to seek, receive, and handle feedback effectively. These include, but not limited to, inaccurate self-assessment, misconception of feedback as a negative act, and negative reaction that may prevent a productive response.

Even if we empower medical educators with the required skills and enhance the educational environment about feedback, medical practices are a busy environment that may limit the feedback process or have an impact on the way it is given. As such, feedback may be missed or delivered in a non-optimal way. Generational differences are also recognized as an important factor in the feedback process and may limit its use. All of the barriers place emphasis on the learner to seek, receive, and then handle feedback efficiently, regardless of whom, when, and how it is delivered. Empowering learners with skills to properly receive feedback will help ensure they gain the benefits from one of the most important tools in education (5, 22).

## Objective

In this review, 10 key tips are provided to help clinical trainees seek, receive, and handle feedback effectively.

Table 1 summarizes the proposed 10 tips to improve receiving feedback.

**Table 1:** Summary of the ten tips

#	Point of emphasis	How to deal with it?
1	Self-assessment	*Break down the task into different components* rather than looking at the *global picture*.
2	Do I really need feedback?	Everyone has a *blind spot*, which prevents us from reaching the next stage of growth, so go and discover it.
3	Your preceptor(s)	*Connect well* with your teacher and build up the bridge of success.
4	Little or no feedback	Take *initiative* and ask for the feedback.
5	Positive feedback	*Thank* your instructor and *appear confident*. Take that task to the proficient level.
6	Your emotion	You are *expected to make mistakes*. It is *normal to receive constructive* feedback. Feedback is an *opportunity for improvement*. Be a good *listener*.
7	Your turn! What after the feedback?	Here is what *really matters*, *be part of* the constructive *action plan* and *follow* that *up*.
8	Generation differences	*Acknowledging* this will help you to better understand your preceptors.
9	General, non-specific feedback	*Probe and ask* questions to figure out what exactly is the point.
10	Be ready for it	Situations matter, feedback can happen at *any time and in any form*.

### Tip #1: Self-assessment

As the initial step in the feedback process, self-assessment is ‘a global judgment of one's ability in a particular domain’ (17). Self-assessment is an integral component of self-regulation and is essential to self-development and educational growth (17, 23). Unfortunately, the literature indicates that you are quite poor at doing this (17, 24). Part of the problem is the way you self-assess by thinking of the big picture (globally) rather than analyzing each step separately. Typically, this type of thinking fails to discover the individual skills of interest. To better assess yourself, try to assess your performance at certain tasks by considering the task as a process and break it down into different components. For instance, when staff ask ‘How things went at a specific patient encounter?’ instead of looking at the global picture, try to dissect the task into different steps, such as building rapport with a patient, history taking, physical examination, and post-encounter discussion. By doing this, your chance to identify an area that needs work is quite high, compared to the global impression.

### Tip #2: We all benefit from feedback

Regardless of where we are in our careers, all of us have blind spots about our abilities that prevent us from reaching the next stage of growth and development. Table 2 illustrates the blind spot issue by using the ‘Johari Window’ (25).

**Table 2:** Johari window

	Known to self	Unknown to self
Known to others	Open	Blind spot
Unknown to others	Hidden	Unknown

In an ideal situation, a small or negligible ‘blind spot’ quadrant is preferable. One way to achieve this is by receiving external feedback openly. You will learn things, either good or bad, about yourself that you were previously unaware of. By acknowledging that individuals are poor at self-assessment, and have a blind spot, you can utilize feedback to help you grow in your career. With that in mind, feedback will provide you with the opportunity to learn of your strengths and weaknesses.

### Tip #3: Connect well with your instructors

Connecting with your instructors is the bridge that promotes the learning process and initiates dialog about your performance. Creating a positive and healthy environment is integral to the feedback process (12). Studies show that preceptors have a weak relationship with students (26, 27). Conversely, the discounting of feedback by students is related to their unfamiliarity with educators (27). Promoting this connection will ease the feedback process and eliminate a major barrier to a successful and powerful tool for self-improvement and development.

### Tip #4: Ask for feedback

Medical practice is quite busy and feedback being overlooked or forgotten has been identified in the literature as one of the barriers to effective feedback (21). Seeking feedback is a powerful initiative towards making improvements. A proactive approach will encourage feedback and from the teacher's perspective, set it as a priority for the student. The teacher will be stimulated to directly observe tasks performed by students, leading to opportunities for productive feedback. Feedback based on observable behavior is identified as a key element for effective and respected feedback (3, 5, 28).

### Tip #5: Be confident and take positive feedback wisely

From clinical practice experience, trainees may have various unproductive reactions to positive feedback ranging from an embarrassing attitude, to fears that something negative will happen and this is just segue way to a negative feedback. Remember, your instructor does pay attention during your clinical practice and observes positive skills and behaviors that need to be continued and built upon in your future career. Appear confident to your instructor by thanking them and be attentive to the details of positive feedback, because this is the basis for growth and development. Successful trainees do not stop when they are good, but instead, take it further and become even more proficient.

### Tip #6: Control your emotions

The feedback process can be emotionally challenging, particularly when negative or unconstructive feedback is given. Whenever you are faced with that, try to think about it as an opportunity for personal growth and development rather than a failure. You are expected to make mistakes, regardless of where you are in your career and sometimes you are not aware of your mistakes. Do not take feedback personally; the focus is not about you, but about the action and what needs to be changed. Be sure to remain calm so you can deal with the feedback. If you are upset, give yourself an opportunity to calm down and first think about the feedback objectively before engaging further.

### Tip #7: Take an action plan

Whether feedback is negative or positive, we immediately try to defend ourselves or rationalize our actions, possibly preventing understanding of the issue and considering a solution. Effective listening is a very powerful skill that allows a clear understanding of the issue and that the teacher is trying to help. It is essential to clarify any issue that appears vague and summarize the main concerns to allow active thinking about an action plan to tackle the issue. What is important about constructive feedback is developing an action plan to change and correct the identified issue (5, 29). Try to develop an action plan that is SMART (specific, measurable, achievable, relevant, and time-bound) (30).

### Tip #8: Acknowledge the generations

The medical field is populated with different generations, and every generation is raised with different ideas and values. Knowing how different generations think and work will enhance your success with the feedback process and to understand how different generations think about feedback. Table 3 illustrates how each generation views feedback.

**Table 3:** Generation and feedback

Generation	Feedback
Traditionalists (1900–1945)	‘No news is good news’.
Baby boomers (1946–1964)	‘Feedback once a year, with lots of documentation!’
Generation X (1965–1980)	‘Sorry to interrupt, but how am I doing?’
Generation Y (1981–1999)	‘Feedback whenever I want it at the push of a button’.

*Note:* Adapted from Lancaster and Stillman (31).

### Tip #9: Be specific and ask about general feedback

Not every clinician is good at providing feedback. Indeed, there is insufficient instruction on how to give feedback (21). A common learner complaint is that feedback is too general, such as ‘good job’ or ‘your performance wasn't great’. Overly general feedback is not helpful (7); however, this does not mean you should dismiss it. Instead, try to probe deeper and find out the actual details of the feedback by asking specific questions as they may lead to a productive conversation about performance.

### Tip #10: Be ready! Feedback is not one type and can be given at any time

Typically, feedback is viewed as formative assessment, occurring halfway during a clinical rotation. Feedback may come at different times and in different formats. Both short and long formats are used in clinical practice. Teachers, healthcare workers, and patients also give feedback. Situations do matter, feedback is dependent on the actual situation, and sometimes immediate feedback is necessary. Even though feedback may take different forms, it usually targets a common goal by reinforcing positive behaviors or correcting performance. The focus needs to be directed to the actual contents of the feedback and not necessarily the format. Acknowledging this will make you ready to receive, and subsequently use the feedback wisely.

## Conclusion

Even with optimizing the delivery of the feedback, learner's self-assessment and receptivity to feedback are essential to completing the successful feedback process and thereby change behaviors and practices. The focus in medical education needs to balance how to deliver feedback with how to receive it. Faculty development efforts need to continue and include learner development efforts. Empowering the learners with skills about how to seek, receive, and handle feedback, regardless of the way it is given, will further improve the feedback process in a busy clinical practice.

The tips provided in this article were intended to provide learners with a framework of strategies to seek, receive, and take feedback to the next level of behavioral and skill changes. The tips are also a useful framework for teaching learners how to be proactive players in the feedback process.
